# Identification of Grb2 protein as a potential mediator of macrophage activation in acute pancreatitis based on bioinformatics and experimental verification

**DOI:** 10.3389/fimmu.2025.1575880

**Published:** 2025-05-26

**Authors:** Qinhao Shen, Shuai Wang, Keyan Wu, Liuhui Wang, Weijuan Gong, Guotao Lu, Weiwei Chen, Chenchen Yuan, Bo Tu, Wei Li, Yaodong Wang, Weixuan Yang

**Affiliations:** ^1^ Pancreatic Center, Department of Gastroenterology, The Affiliated Hospital of Yangzhou University, Yangzhou University, Yangzhou, China; ^2^ Department of Gastroenterology, Huai’an Hospital Affiliated to Yangzhou University (The Fifth People’s Hospital of Huai’an), Yangzhou University, Huai’an, China; ^3^ Department of Gastroenterology, Clinical Medical College, Yangzhou University, Yangzhou, China; ^4^ Clinical Research Division, Fred Hutchinson Cancer Research Center, Seattle, WA, United States; ^5^ Faculty of Pharmaceutical Sciences, Toho University, Funabashi, Chiba, Japan; ^6^ Department of Gastroenterology, Kunshan Hospital of Traditional Chinese Medicine, Suzhou Key Laboratory of Integrated Traditional Chinese and Western Medicine of Digestive Diseases, Kunshan Affiliated Hospital of Yangzhou University, Kunshan, China

**Keywords:** GRB2, acute pancreatitis, macrophage activation, scRNA-seq, inflammation

## Abstract

**Introduction:**

Macrophage activation is closely associated with Acute pancreatitis (AP). We screened and found that Growth factor receptor bound protein 2 (Grb2) is highly expressed in macrophages during AP. However, the relationship between Grb2 and AP is still poorly understood. In this study, we explored the role of Grb2 in AP.

**Methods:**

We screened for gene affecting macrophage activation in AP by combining transcriptomics with Single-cell RNA-sequence analysis. Next, the expression of Grb2 in M1/M2 macrophage activation was detected by Single-cell RNA-sequence analysis and western blot. Furthermore, the effect of Grb2 on M1/M2 macrophage activation was detected by flow cytometry. The severity of AP was assessed by histological analysis, serum amylase, serum lipase and serum inflammatory factors *in vivo*. NOD-like receptor thermal protein domain associated protein 3 (Nlrp3) and Nuclear factor kappa-B (NF-kB) signaling pathways were also evaluated.

**Results:**

Grb2 is mainly expressed in macrophages of pancreas in AP and up-regulated in M1 macrophage activation. Inhibiting Grb2 could alleviate AP by preventing M1 macrophage activation through down-regulating Nlrp3 and NF-κB.

**Discussion:**

Inhibition of Grb2 can effectively prevent M1 macrophage activation and alleviate AP. Grb2 may potentially be an effective target of macrophage activation for the treatment of AP.

## Introduction

AP is a prevalent gastrointestinal disease marked by acinar cell death and the infiltration of inflammatory cells (1, 2). The incidence of AP is increasing globally. Mounting evidence suggest that the infiltration of inflammatory immune cells impacts the severity of AP. The inflammatory cells infiltrated in acute pancreatitis are predominantly neutrophils and macrophages (3–6). Neutrophil forming Neutrophil Extracellular Traps (NETs) can lead to pancreatic tissue damage (7, 8). The activated macrophages in AP are mainly classified into two types. The macrophages infiltrating in the early stage of AP are generally pro-inflammatory M1 macrophage, while anti-inflammatory M2 macrophages typically play a role in the repair period of pancreatic tissue (9, 10). Hence, targeting macrophage activation could be a strategy for the treatment of AP.

Grb2 is a non-enzymatic adaptor protein consisting of a single Src homology 2 (SH2) domain and two Src homology 3 (SH3) domains (11, 12). The classic Grb2 signaling pathway is initiated by receptor tyrosine kinases (RTKs) and plays an important role in cell signal transduction. Studies have indicated that Grb2 plays a crucial role in cell proliferation (13–15),cell death (16–19) and cell differentiation (20, 21). In addition, Grb2 is closely related to immune regulation or inflammatory diseases. Treg cell-specific Grb2 deletion abrogates the augmentation of allergic airway inflammation by Il4ra^R576^ (22). In the inflammatory lesions of the infected liver, Grb2 was positive in both hepatocytes and infiltrating leukocytes (23). The expression of GRB2 was increased in psoriatic lesions compared with normal skin adjacent to psoriasis and healthy controls. Inhibition of GRB2 inhibits the secretion of inflammatory mediators in keratinocytes (24).In this research, Grb2 was primarily expressed in macrophages of pancreas in AP. Inhibition of Grb2 can prevent the transformation of macrophage into M1 macrophage and alleviated pancreatic tissue injury caused by caerulein-induced AP. Thus, Grb2 may become an potential target of macrophage activation in AP.

## Experimental methods

### Antibodies and reagents

All reagents and antibodies involved in this study are shown in **Supplementary Tables S1**, **S2**.

### Animals

C57BL/6 mice were purchased from GemPharmatech Co., Ltd. (Nanjing, China). All mice were raised in a suitable environment (20-25°C, 12h light/12h dark) without specific pathogen. They were provided with sufficient water and food. All studies were approved by Yangzhou University (No.202307018).

### Construction of the AP model and Grb2 inhibitors administration

Caerulein (Cae) model, acute pancreatitis model was established by intraperitoneal (i.p.) injection of Caerulein (100 mg/kg, 1h interval, 10 times). At 0 hour, GRB2 inhibitor prexigebersen (20 mg/kg, 40 mg/kg, 80mg/kg) was administered via intravenous (i.v.) injection. 12 hours later, mice were anesthetized and euthanized. Serum samples were collected for serological analysis and ELISA assays. Pancreatic tissues were harvested and stored at -80°C for subsequent experiments.

Pancreatic duct ligation (PDL)-induced AP model: All mice were anesthetized and placed on a horizontal surgical table. A longitudinal incision of 1–2 centimeters was made on the abdomen to expose the abdominal cavity. The duodenum was turned over to expose the pancreatic duct. At a position 1 centimeter above the duodenal papilla, the tissue around the pancreatic duct was bluntly dissected, and the pancreatic duct was ligated with a ligation thread. The silk thread completely blocked it to simulate cholestasis and biliary pancreatitis caused by cholelithiasis. Then, the abdominal cavity was sutured in layers. At 1 hour, GRB2 inhibitor prexigebersen (40 mg/kg) was administered via intravenous (i.v.) injection. 24 hours later, mice were anesthetized and euthanized.

Serum samples and pancreatic tissues were harvested or subsequent experiments.

### ELISA assays

Following the manufacturer’s protocol, the plates were coated with antibody, sealed, and incubated overnight at 4°C. The following day, the liquid was aspirated, and the plates were washed three times with phosphate-buffered saline containing 0.05% Tween 20 (PBST). Blocking was performed using ELISPOT blocking buffer for 1 h at room temperature (RT). After removing the blocking solution and washing with PBST, serum samples and standards were added to the plates. The plates were sealed and incubated at RT for 2 h. Subsequently, the liquid was discarded, and the plates were washed three times with PBST. Detection Antibody (diluted according to the datasheet) was added, followed by sealing and incubation at RT for 1 h. After additional PBST washes, Streptavidin-HRP conjugate (1:250 dilution) was introduced, and the plates were sealed and incubated in the dark at RT for 30 min. Following further PBST washing, TMB Substrate Solution was added, and the plates were incubated in the dark at RT for 15 min. The reaction was terminated by adding Stop Solution. Absorbance was measured at 450 nm using a microplate reader.

### Extraction and induction of bone marrow-derived macrophages (BMDMs)

The leg bones of the mice were isolated and soaked in 75% alcohol for 1 min. Subsequently, the bone marrow cavity was washed with 1640 medium. The cells were collected after centrifugation at 500g for 5 minutes. Macrophages were cultured in 1640 medium containing 20ng/ml macrophage-stimulating factor (M-CSF), 10% fetal bovine serum (FBS), 1% penicillin and 1% streptomycin for 7 days in 37°C, 5% CO_2_ incubator. The medium was changed every other day and mature macrophages were obtained after 7 days.

### Flow cytometry

Mature BMDMs were stimulated with LPS (100ng/ml) and INFγ (10ng/ml) for 24h.The BMDMs were incubated with flow cytometry antibody F4/80 (1:200 dilution) and CD86 (1:200 dilution), washed with PBS and resuspended with PBS. M1 activation of BMDM was detected by flow cytometry. Mature BMDMs were stimulated with IL-4 (50ng/ml) for 24h, incubated with flow cytometry antibody F4/80 (1:200 dilution) and CD206 (1:200 dilution) at 4°C for 30min, washed with PBS and resuspended with PBS. M2 activation of BMDM was detected by flow cytometry.

### Western blot

RIPA lysis buffer and protease inhibitor (1:10) were mixed to lyse BMDMs. The BMDMs were sonicated, centrifuged at 12000 rpm at 4°C for 30 min. The protein supernatant was collected, added protein loading buffer, and boiled at 100°C for 10 min. The protein samples were subjected to SDS-PAGE, transferred to PVDF membrane. The membranes were blocked with 5% skim milk for 2 h, washed with TBST (3 times, 5 min each time), incubated with primary antibody (1:1000 dilution) overnight at 4°C. The next day, the membranes were washed with TBST (3 times, 15min each time), and incubated with second antibody(1:5000 dilution) at room temperature for 2 h. TBST washing (3 times, 10min each time). The protein bands were detected by ECL Luminometer and ImageJ was used to analyze the gray value.

### Cell siRNA transfection

For one well of a 24-well plate: Diluted 2 μL Lipofectamine 3000 (Lipo3000) in 50 μL serum-free RPMI 1640 medium, mixed gently, and incubated at room temperature for 5 minutes. Diluted 4 μL siRNA in 50 μL serum-free RPMI 1640 medium, then combined this mixture with the prepared Lipo3000/1640 mixture. Mixed thoroughly and incubated at room temperature for 20 minutes to allow complex formation. Added the resulting transfection complex mixture to the cell culture plate. After 24 hours, replaced the medium with fresh RPMI 1640 medium supplemented with 10% FBS. Performed subsequent experiments 48 hours post-transfection.

### Histological analysis

Pancreatic tissue samples were fixed in 4% paraformaldehyde, paraffin-embedded, and sectioned into 5-μm-thick slices. After deparaffinization and dehydration, the sections were stained with hematoxylin and eosin (H&E). Histopathological examination was performed using a light microscope. The severity of acute pancreatitis was evaluated according to previously established methodologies (25).

### Immunofluorescence

The slices were treated with dewaxing, dehydration, membrane rupture, antigen repair, blocking. The slices were incubated with primary antibody (1:100 dilution) overnight at 4°C. On the second day, the slices were washed with PBS (3 times, 5 minutes each), incubated with secondary antibody (1:1000 dilution) at room temperature for 2 hours and washed again with PBS (3 times, 5 minutes each). Added DAPI and anti-quenching agent, then sealing. The results were detected by laser scanning confocal microscope (Nikon, Ti2-E-A1 HD25).

### Isolation of Peripheral blood mononuclear cells (PBMCs)

The anticoagulated blood was layered over Ficoll separation medium, and density gradient centrifugation was performed. The PBMC layer was collected. The cells were washed with PBS, centrifuged to remove the supernatant, and lysed with lysis buffer to extract proteins.

### Microarray analysis

The microarray data of AP GSE65146, GSE109227 were downloaded from Gene Expression Omnibus (GEO) database (26). Both data had been log2 transformed. Limma package (27) was employed for identifying differentially expressed genes (DEGs). All genes (|log2FoldChange|>0) were analyzed by Gene Set Enrichment Analysis (GSEA) via clusterProfiler package (28, 29).

### RNA-seq analysis

The RNA-seq data of macrophage GSE138134 (9) and RNA-seq data of M1 macrophage activation GSE247106 (30) were downloaded from GEO database. The DEGs (log2FoldChange>0.5, padj<0.01) were analyzed by DESeq2 package (31) or edgeR package (32–34). The pheatmap package was employed to display DEGs.

### Single-cell RNA-sequence analysis

The scRNA-seq data of AP GSE188819 (35) and scRNA-seq data of macrophage activation GSE117176 (36),GSE158094 (37) were downloaded from GEO database. Downstream analysis was performed using Seurat package (V5) (38).

GSE188819: Following quality control (QC), cells with fewer than 200 expressed genes and >10% mitochondria-related genes were excluded. Harmony R package (39) was used to eliminate batch effects between different samples. As a result, a SeuratObject with 31080 cells and 22508 genes was subsequently normalized and scaled for the subsequent analysis, including RunPCA and RunTSNE. The cells were then clustered using the FindNeighbors and FindClusters functions with a resolution of 0.4. The generated clusters were visualized using T-distributed stochastic neighbor embedding (tSNE) plot.

GSE117176: Following quality control (QC), cells with fewer than 200 expressed genes and >10% mitochondria-related genes were excluded. Merge different samples. As a result, a SeuratObject with 17661 cells and 15217 genes was subsequently normalized and scaled for the subsequent analysis, including RunPCA and RunTSNE. The cells were then clustered using the FindNeighbors and FindClusters functions with a resolution of 0.1. The generated clusters were visualized using T-distributed stochastic neighbor embedding (tSNE) plot. FindMarkers was used to analyze DEGs (log2FoldChange>0.5, padj<0.01).

GSE158094: Three samples (24h_M0, 24h_M1 and 24h_M2) were selected for analysis. Following quality control (QC), cells with fewer than 200 expressed genes and >10% mitochondria-related genes were excluded. Merge different samples. As a result, a SeuratObject with 8492 cells and 12523 genes was subsequently normalized and scaled for the subsequent analysis, including RunPCA and RunTSNE. The cells were then clustered using the FindNeighbors and FindClusters functions with a resolution of 0.1. The generated clusters were visualized using T-distributed stochastic neighbor embedding (tSNE) plot. FindMarkers was used to analyze DEGs (log2FoldChange>0.5, padj<0.01).

Spearman Correlation Analysis was performed to assess the relationship between the two genes. KEGG (Kyoto Encyclopedia of Genes and Genomes) enrichment analysis were performed to analyze Genes positively associated with Grb2.

### Short time-series expression miner analysis

The detection of gene clustering profiles were conducted by using the STEM (40, 41) clustering algorithm to identify temporal gene expression profiles, with the maximum number of model profiles set to 50 and maximum unit change in model profiles between time points set to 3.

### Statistical analysis

Statistical analysis was performed by GraphPad Prism 8 software (GraphPad, San Diego, CA, USA) and the results were presented as mean ± standard deviation (SD). The difference between two groups was analyzed by t-test, and the difference between more than two groups was analyzed by one-way ANOVA test. P < 0.05 was considered statistically significant (two-tailed).

## Results

### Grb2 is mainly expressed in pancreatic tissue macrophages in AP

**Figure 1:**
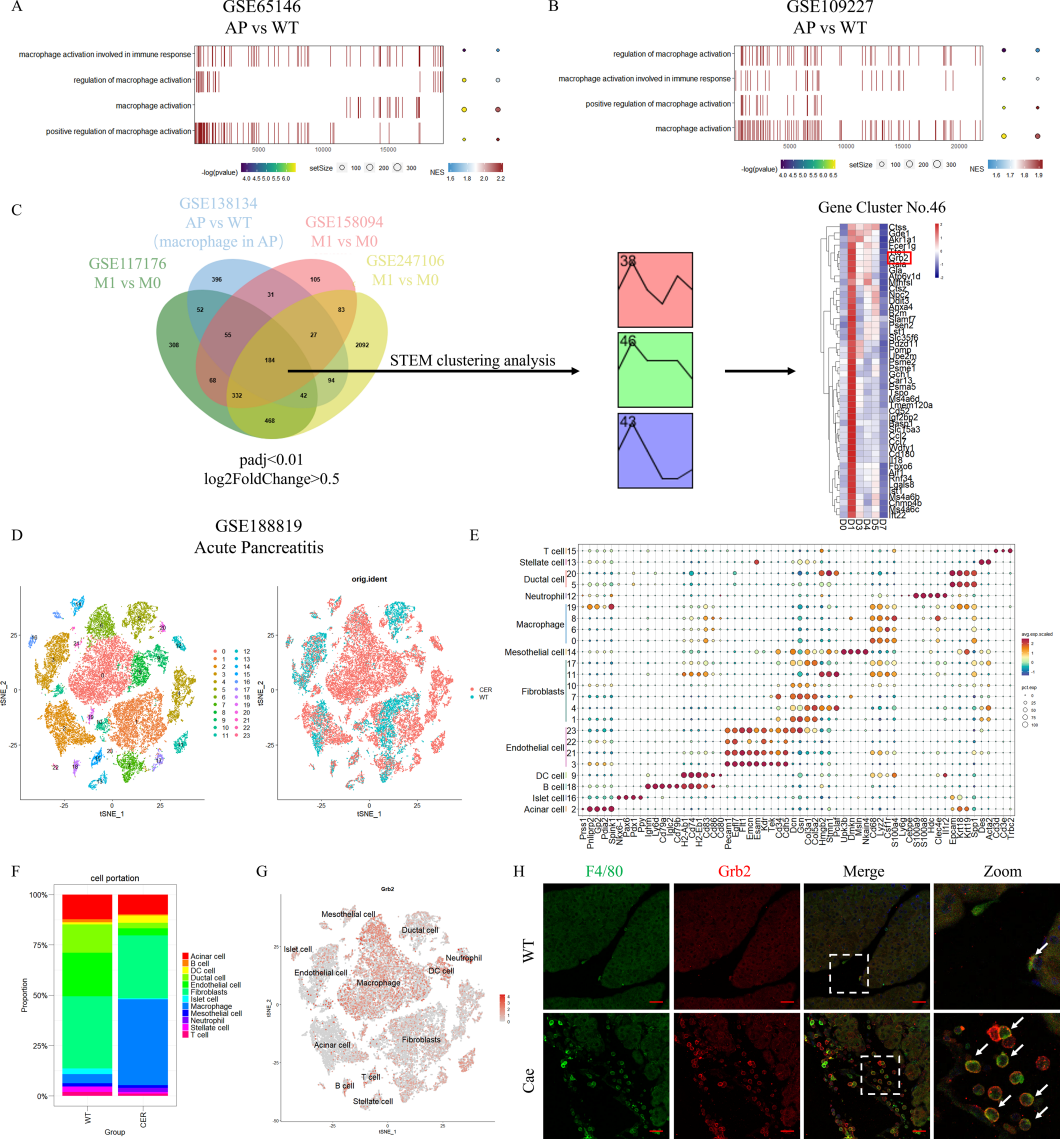
Grb2 is mainly expressed in macrophages of pancreas in AP. **(A,B)** GSEA pathway enrichment analysis of AP microarray data GSE65146 and GSE109227. (AP versus WT). **(C)** The flowchart of screening differentially expressed genes. **(D)** The t-distributed Stochastic Neighbor Embedding (tSNE) visualization of AP scRNA-seq GSE188819.**(E)** DotPlot showing the expression of marker genes for each annotated cell type: T cell (Cd3d, Cd3e,Trbc2), Stellate cell (Des, Acta2), Ductal cell (Epcam, Krt18, Krt19, Spp1), Neutrophil (Ly6g, Cebpe, S100a9, S100a8, Hdc, Clec4e, Il1r2), Macrophage (Cd68, Lyz2, Csf1r, S100a4), Mesothelial cell (Upk3b, Dmkn, Msln, Nkain4), Fibroblasts (Dcn, Gsn, Col3a1, Col5a2, Hmgb2, Stmn1, Pclaf), Endothelial cell (Pecam1, Egfl7, Flt1, Emcn, Esam, Kdr, Tek, Cd34, Cdh5), DC cell (H2-Ab1, Cd74, H2-Eb1, Cd83, Cd86, Cd80), B cell (Ighm, Ly6d, Cd79a, Iglc2, Cd79b), Islet cell (Nkx6-1, Pax6, Pdx1, Ppy), Acinar cell (Prss1, Pnliprp2, Gp2, Pdia2, Spink1). **(F)** The cell proportion of cells derived from different cell type. **(G)** The tSNE visualization of the expression of Grb2. **(H)** Representative Immunofluorescence images for Grb2 and F4/80 expression in macrophage of AP mice. The white arrow represents colocalization of Grb2 and F4/80. Scale Bar = 50μM.

According to the results of GSEA of pancreatic tissue microarray data of AP (GSE65146 and GSE109227), it was found that macrophage activation involved in immune response, regulation of macrophage activation, macrophage activation, positive regulation of macrophage activation were significantly up-regulated in AP (**Figures 1A, B** and **Supplementary Table S3**, **S4**). This indicates that macrophages in pancreas are activated when AP occurs.

We conducted a joint analysis of RNA-seq and scRNA-seq datasets (GSE117176, GSE138134, GSE158094 and GSE247106). As a result, we found a total of 184 common up-regulated DEGs. Next, a total of 3 clusters were screened by STEM clustering analysis (**Supplementary Table S5**). Among them, cluster 46 gene expression increased in the early stage and gradually decreased to return to the baseline level. We screened Grb2 for subsequent experiments through heatmap analysis (**Figure 1C**).

We obtained 24 cell clusters through cell clustering analysis on AP scRNA-seq data (GSE188819) (**Figure 1D**). Then we annotated different clusters based on cell-specific, highly expressed genes (**Figure 1E** and **Supplementary Table S6**). Next, we carried out cell proportion analysis. The number of macrophages increased significantly during AP (**Figure 1F**). FeaturePlot demonstrated that Grb2 was mainly expressed in pancreatic macrophages (**Figure 1G**). The results of immunofluorescence were consistent with those of scRNA-seq (**Figure 1H**). In addition, we detected PBMC in peripheral blood of AP patients and found that Grb2 was up-regulated in PBMC of AP patients compared with PBMC of healthy people (**Supplementary Figures S1A, B**). Therefore, we speculated that Grb2 may play a role in macrophage activation during AP.

### Grb2 is up-regulated in M1 macrophage activation

**Figure 2:**
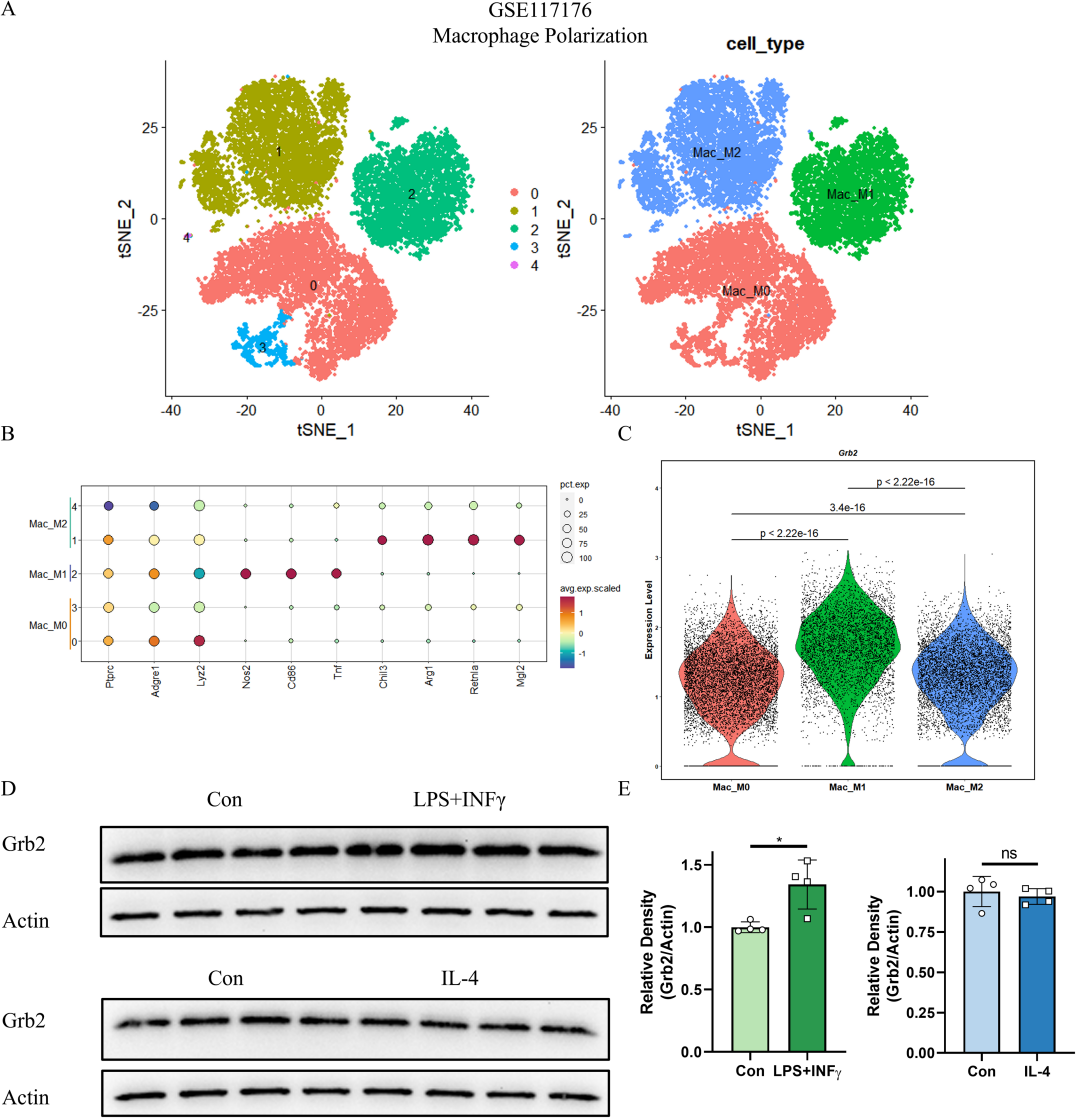
Grb2 is up-regulated in M1 macrophage activation. **(A)** The tSNE visualization of macrophage activation scRNA-seq GSE117176. **(B)** DotPlot of the three major cell types (Columns) and their marker genes (Rows). **(C)** VlnPlot showed the expression of Grb2. **(D)** Protein levels of Grb2 in macrophage activation were analyzed by western blotting. **(E)** Relative density of Grb2.Beta-actin was used as control for protein loading, N=4 each group. *P < 0.05. LPS, Lipopolysaccharide; INFγ, Interferon-γ; IL-4, Interleukin 4.

To verify the role of Grb2 in macrophage, we initially analyzed the scRNA-seq data of macrophage activation (GSE117176) and obtained 5 cell clusters (**Figure 2A**). Subsequently, we performed cell annotation. M1 macrophages highly expressed Nos2, Cd86, Tnf and M2 macrophages highly expressed Chil3, Arg1, Retnla, Mgl2. M0 macrophages only expressed Ptprc, Adgre1, Lyz2, and did not express M1M2-specific genes. (**Figure 2B**). Nos2, Cd86 and Tnf were mainly expressed in M1 macrophage, while Chil3, Arg1, Retnla and Mgl2 were highly expressed in M1 macrophage (**Figure 2B**). The VlnPlot demonstrated that Grb2 was up-regulated in M1 macrophages and M2 macrophages (**Figure 2C**). Western blot results showed expression of Grb2 was up-regulated in M1 macrophages, but not in M2 macrophages. (**Figures 2D, E**). Thus, we hypothesized that Grb2 only exerts an effect on M1 macrophage activation.

### Grb2 is associated with immune and inflammatory pathways

**Figure 3:**
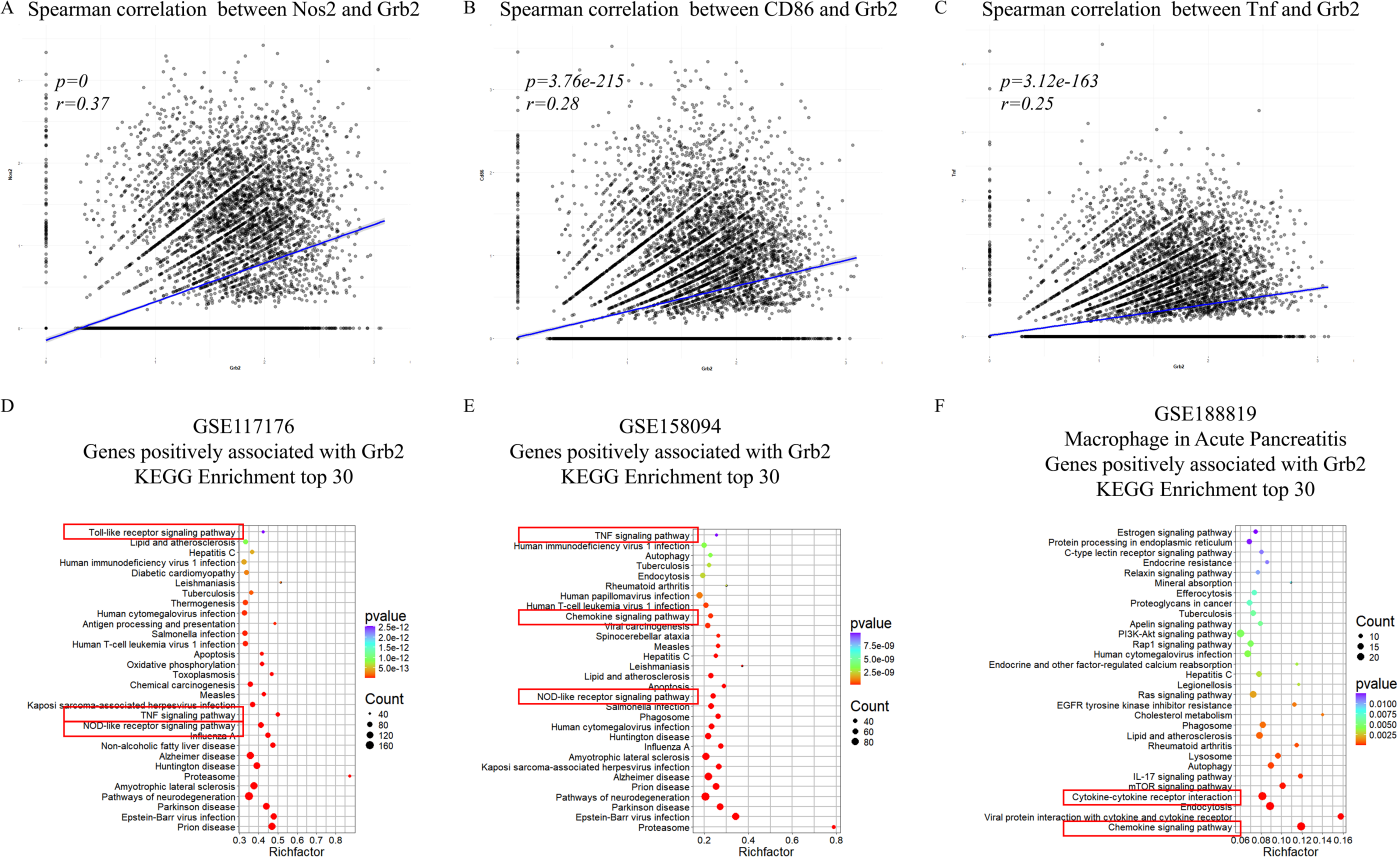
Grb2 is associated with immune and inflammatory pathway. **(A)** The Spearman correlation between Nos2 and Grb2. **(B)** The Spearman correlation between CD86 and Grb2. **(C)** The Spearman correlation between Tnf and Grb2. **(D)** KEGG analysis of genes positively associated with Grb2 of macrophage activation scRNA-seq GSE117176. **(E)** KEGG analysis of genes positively associated with Grb2 of macrophage activation scRNA-seq GSE158094. **(F)** KEGG analysis of genes positively associated with Grb2 of AP scRNA-seq GSE188819.

To verify the relationship between and macrophage activation. In GSE117176, correlation analysis revealed that Grb2 was positively correlated with M1 macrophage activation marker Nos2, CD86 and Tnf (**Figures 3A–C**), but not with M2 macrophage activation markers Chil3, Arg1, Retnla and Mgl2 (**Supplementary Figures S2A-D**). Kyoto Encyclopedia of Genes and Genomes (KEGG) analysis of genes positively related to Grb2 in the scRNA-seq data of M1 macrophage activation (GSE117176 and GSE158094) demonstrated that Grb2 was associated with immune and inflammatory pathways such as the NOD-like receptor signaling pathway and the TNF signaling pathway (**Figures 3D, E**). Additionally, in the scRNA-seq data of AP (GSE188819), Grb2 was also found to be related to immune and inflammatory pathways such as the Chemokine signaling pathway and the Cytokine-cytokine receptor interaction (**Figure 3F**).

### Inhibition of Grb2 inhibits M1 macrophage activation

**Figure 4:**
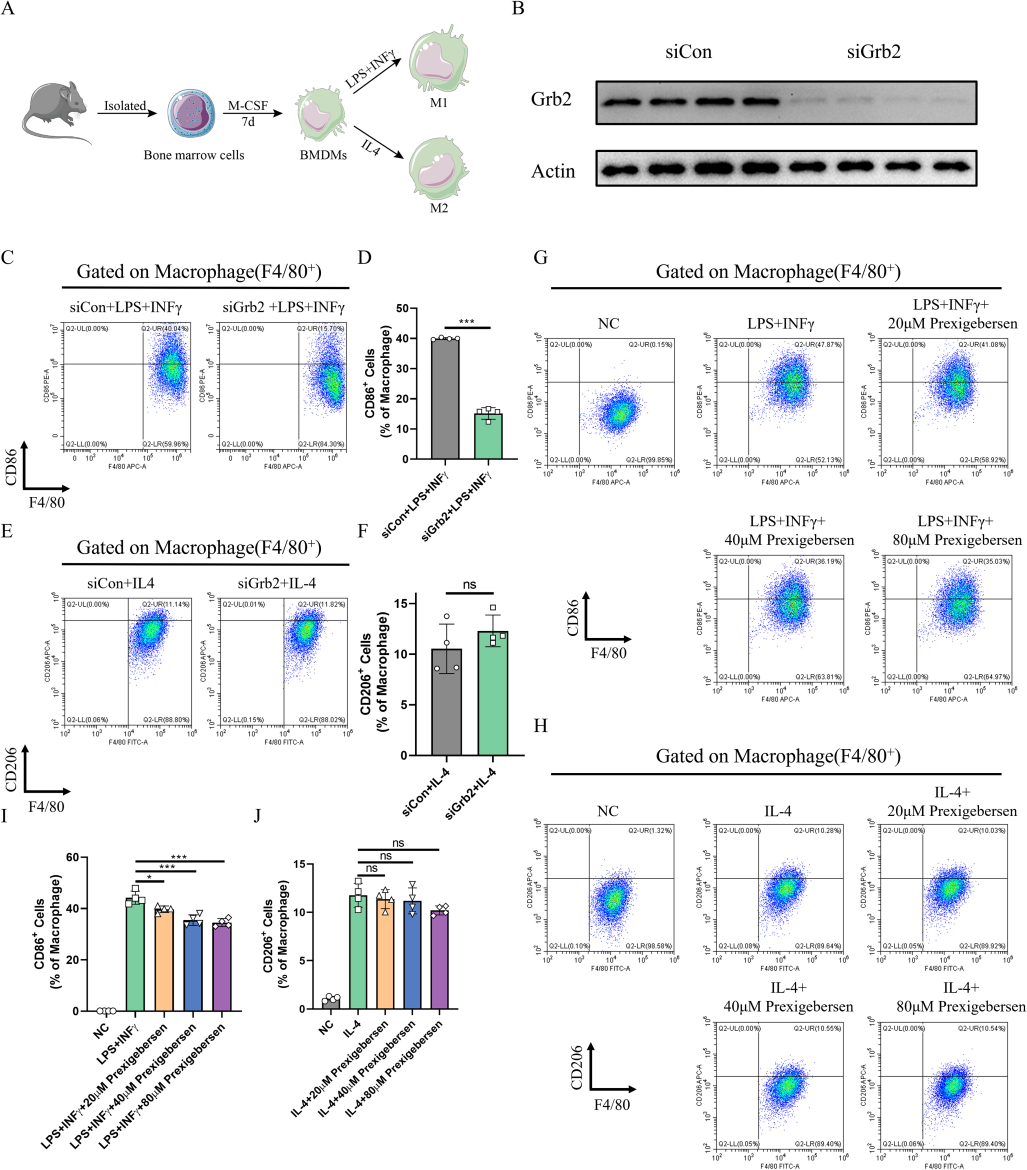
Inhibition of Grb2 inhibits M1 macrophage activation. **(A)** The flowchart of macrophage activation. **(B)** The silencing efficiency of Grb2 was analyzed by western blotting. **(C, D)** CD86 (M1 macrophage marker) and F4/80 (macrophage marker) expression levels in macrophages treated with Grb2 siRNA were detected by flow cytometry, N=4 each group. **(E, F)** CD206 (M2 macrophage marker) and F4/80 (macrophage marker) expression levels in macrophages treated with Grb2 siRNA were detected by flow cytometry, N=4 each group. **(G)** CD86 (M1 macrophage marker) and F4/80 (macrophage marker) expression levels in macrophages treated with Grb2 inhibitor prexigebersen were detected by flow cytometry, N=4 each group. **(H)** CD206 (M2 macrophage marker) and F4/80 (macrophage marker) expression levels in macrophages treated with Grb2 inhibitor prexigebersen were detected by flow cytometry, N=4 each group. **(I, J)** Quantification of M1/M2 macrophage activation detected by flow cytometry. *P < 0.05 and ***P < 0.001.

To clarify the role of Grb2 in macrophage activation, BMDMs were extracted to induce M1/M2 macrophage activation (**Figure 4A**). SiRNA was employed to silence the expression of Grb2 in BMDMs, and western blot was utilized to verify the silencing efficiency (**Figure 4B**). The silencing of Grb2 significantly inhibited M1 macrophage activation but had no significant impact on M2 activation (**Figures 4C–F**). Subsequently, the Grb2 inhibitor prexigebersen was used, and it was found that the results of the Grb2 inhibitor prexigebersen were consistent with those of Grb2 silencing (**Figures 4G–J**). All these results confirmed that Grb2 affected M1 macrophage activation and had little effect on M2.

### Grb2 inhibitor alleviates AP *in vivo*

**Figure 5:**
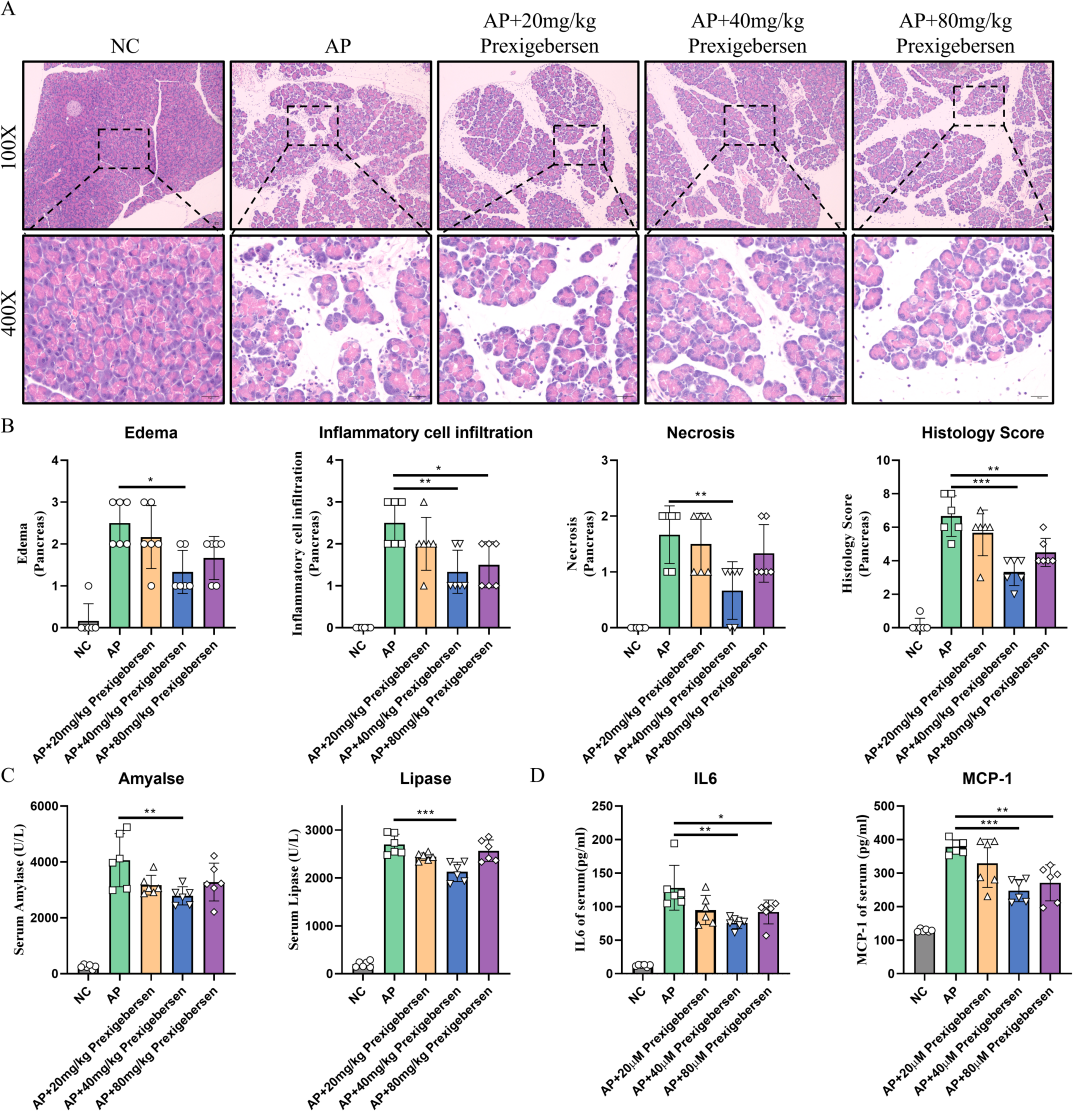
Grb2 inhibitor alleviates Cae-induced AP. **(A)** Representative HE staining of pancreatic tissues in magnifications 100x and 400x. Scale Bar = 50μM. **(B)** The pathological scores of pancreatic tissues. **(C)** Serum levels of amylase and lipase, N=6 each group. **(D)** The serum levels of IL-6, MCP-1 were detected by ELISA. N= 6 each group. *P < 0.05, **P < 0.01 and ***P < 0.001.

To explore the role of Grb2 *in vivo*, we established a classic acute pancreatitis model using caerulein and treat with the Grb2 inhibitor prexigebersen at 0h. In the Cae group, the pancreas of mice showed edema, inflammatory cell infiltration and cell necrosis. In the medium-dose prexigebersen treatment group, the edema, inflammatory cell infiltration and cell necrosis of pancreas of mice were significantly improved (**Figures 5A, B**). Additionally, the levels of serum amylase and lipase levels in the medium-dose Prexigebersen group were lower than those in the Cae group (**Figures 5C, D**). We detected serum inflammatory factors Interleukin-6 (IL-6) and Monocyte Chemoattractant Protein-1 (MCP-1) in mice, reflecting inflammation in mice. IL-6 and MCP-1 were significantly increased in AP mice. Grb2 Inhibitor prexigebersen reduces serum IL-6 and MCP-1 levels (**Figure 5D**). We further verified in another PDL model, and the results showed that Grb2 inhibitor prexigebersen also alleviated the severity of PDL-induced acute pancreatitis (**Supplementary Figures S3A-C**). All these results indicate that the Grb2 inhibitor prexigebersen alleviates AP *in vivo*.

### Grb2 inhibitor inhibits Nlrp3 and NF-kB in macrophages

**Figure 6:**
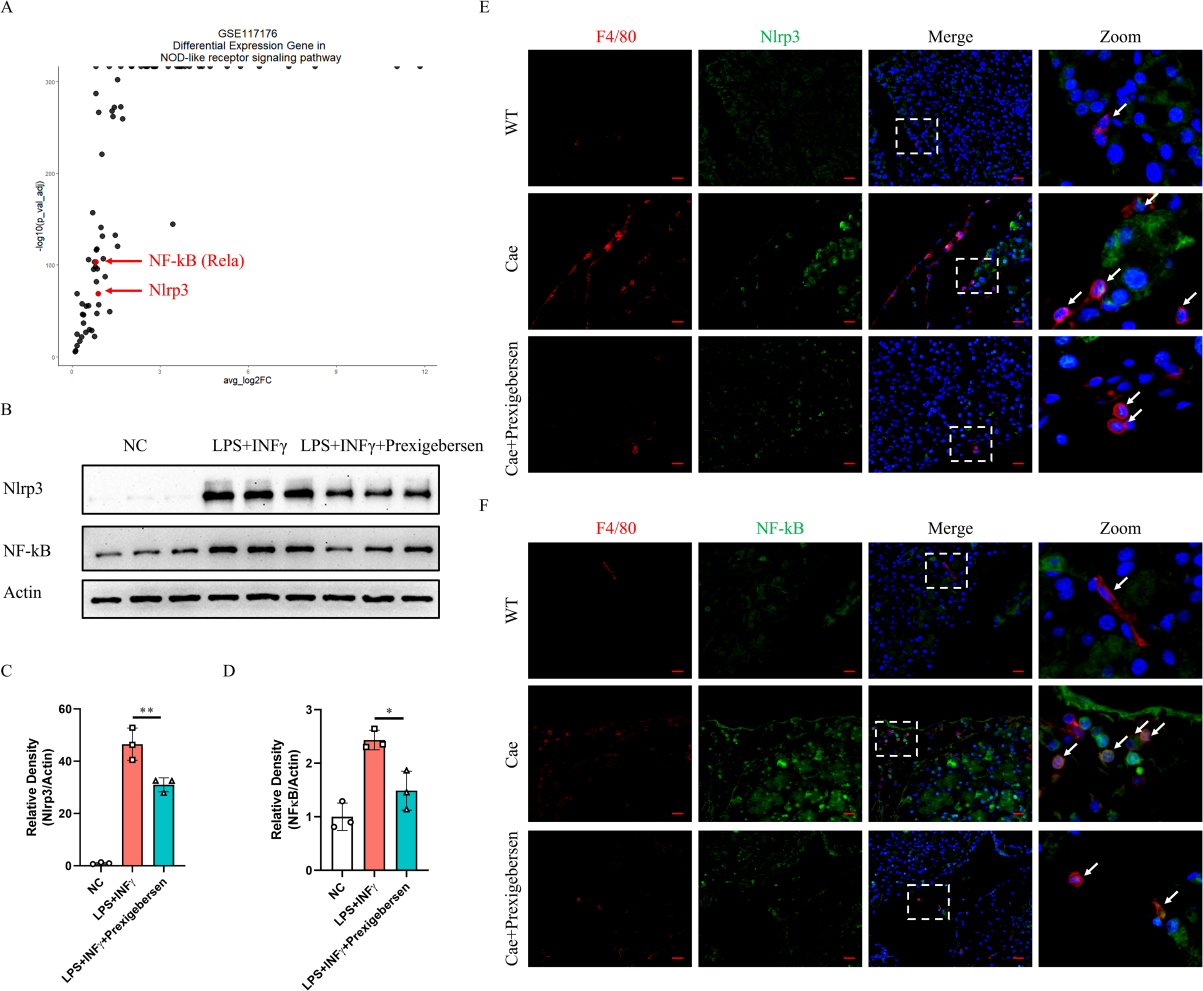
Nlrp3 and NF-κB were inhibited by the Grb2 inhibitor in macrophages of AP mice. **(A)** The volcano plot of Differential Expression Gene in NOD-like receptor signaling pathway of GSE117176. **(B)** Protein levels of Nlrp3 and NF-kB in M1 macrophage activation were analyzed by western blotting. **(C, D)** Relative density of Nlrp3 and NF-kB. Beta-actin was used as control for protein loading, N=3 each group. *P < 0.05, **P < 0.01. **(E)** Representative Immunofluorescence images for Nlrp3 and F4/80 expression in macrophage of AP mice. **(F)** Representative Immunofluorescence images for NF-kB and F4/80 expression in macrophage of AP mice. Scale Bar = 20μM.

To further investigate the downstream mechanism of Grb2, we discovered that Nlrp3 and NF-kB (Rela) were significantly up-regulated in the NOD-like receptor signaling pathway (**Figure 6A**). Recently, increasing evidence has suggested that Nlrp3 and NF-kB play a crucial role in M1 macrophage activation (42–45). We observed that Nlrp3 and NF-kB were upregulated during M1 macrophage activation *in vitro*. The Grb2 inhibitor prexigebersen reversed the upregulation of Nlrp3 and NF-kB (**Figures 6B–D**). Consequently, we performed immunofluorescence analysis of pancreas of AP mice. The results demonstrated that Nlrp3 and NF-kB were up-regulated in macrophages during AP, and Grb2 inhibitor prexigebersen inhibited the expression of Nlrp3 and NF-KB in macrophage (**Figures 6E, F**). The above results indicate that Grb2 may influence M1 macrophage activation by regulating Nlrp3 and NF-kB.

## Discussion

It is widely acknowledged that macrophages are one of essential cells during AP inflammation (9, 46–48). Through joint analysis of multiple RNA-seq and scRNA-seq databases, we discovered that Grb2 was highly expressed in macrophages during AP. Researchers have found that inhibition of Grb2 repolarized alveolar macrophages from M1 to M2 phenotype (49). Consequently, we hypothesize that Grb2 may play a role in AP by regulating macrophage activation.

We discovered up-regulation of Grb2 in M1 macrophage activation through single-cell RNA-sequence analysis, whereas there was no significant alteration in M2. The result was validated by western blot. Subsequently, we further investigated the function of Grb2. Inhibition of Grb2 hindered M1 macrophage activation, but had no impact on M2. M1 macrophage activation affects AP. Inhibition of M1 macrophage activation can effectively mitigate AP (42, 50, 51). To study the effect of Grb2 in the pathological process of AP, we employed the Grb2 inhibitor prexigebersen to treat caerulein-induced AP mice and found that prexigebersen alleviated the severity of AP and decreased the inflammatory response in AP mice.

A number of studies have demonstrated that Nlrp3 and NF-kB play a crucial role in M1 macrophage activation (52–55). We discovered that Nlrp3 and NF-kB were up-regulated in M1 macrophage activation. Both *in vitro* and *in vivo* experiments have proved that Nlrp3 and NF-kB were significantly up-regulated in M1 macrophage activation or macrophages of AP group, and Grb2 inhibitor prexigebersen inhibited the expression of Nlrp3 and NF-kB in macrophages. In summary, Inhibition of Grb2 can alleviate AP by down-regulating Nlrp3 and NF-kB of macrophage.

## Conclusion

Overall, our study firstly demonstrates that inhibition of Grb2 can effectively prevent M1 macrophage activation and alleviate AP. Grb2 may potentially be an effective target of macrophage activation for the treatment of AP.

## Data Availability

The original contributions presented in the study are included in the article/**Supplementary Material**. Further inquiries can be directed to the corresponding authors.

## Supplementary material

Supplementary Table 1 Reagent table.

Supplementary Table 2 Antibody table

Supplementary Table 3 The list of genes for pathways of GSE65146

Supplementary Table 4 The list of genes for pathways of GSE109227

Supplementary Table 5 The list of genes for cluster of STEM clustering analysis

Supplementary Table 6 Markers for identifying cell types.

Supplementary Figure 1 GRB2 is upregulated in PBMC of AP patients. **(A)** Protein levels of GRB2 in PBMC were analyzed by western blotting. **(B)** Relative density of Grb2.Beta-actin was used as control for protein loading, N=4 each group. *P < 0.05.

Supplementary Figure 2 Grb2 is not associated with M2 marker. **(A)** The Spearman correlation between Chil3 and Grb2. **(B)** The Spearman correlation between Arg1 and Grb2. **(C)** The Spearman correlation between Retnla and Grb2. **(D)** The Spearman correlation between Mgl2 and Grb2.

Supplementary Figure 3 Grb2 inhibitor alleviates PDL-induced AP. **(A)** Representative HE staining of pancreatic tissues in magnifications 100x and 400x. Scale Bar = 50μM. **(B)** Serum levels of amylase and lipase. **(C)** The pathological scores of pancreatic tissues. N=6 each group. N= 6 each group. *P < 0.05, **P < 0.01.
