# Supplementary figures and images for: Identification of Grb2 protein as a potential mediator of macrophage activation in acute pancreatitis based on bioinformatics and experimental verification
Source: Front Immunol. 2025 May 26;16:1575880. doi: 10.3389/fimmu.2025.1575880 (PMC12146204; doi:10.3389/fimmu.2025.1575880)

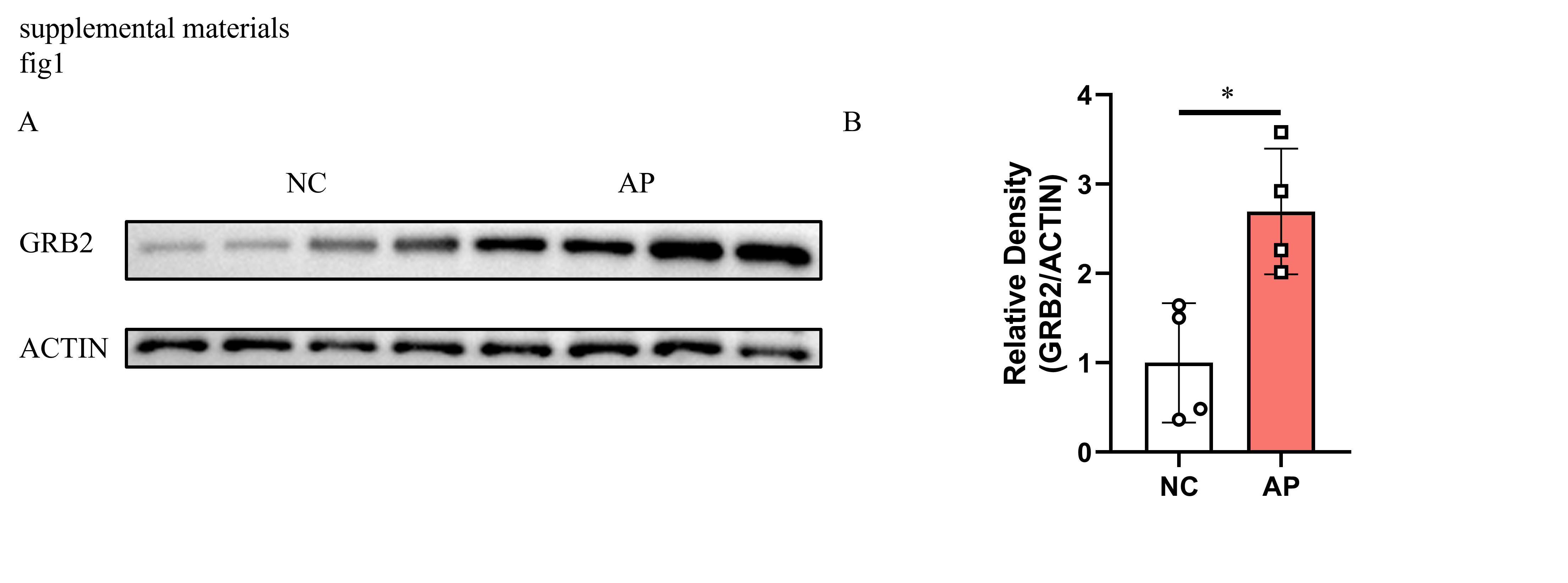

Supplement: Supplementary Figure 1 — GRB2 is upregulated in PBMC of AP patients. (A) Protein levels of GRB2 in PBMC were analyzed by western blotting. (B) Relative density of Grb2.Beta-actin was used as control for protein loading, N=4 each group. *P < 0.05. [file Image1.tif]

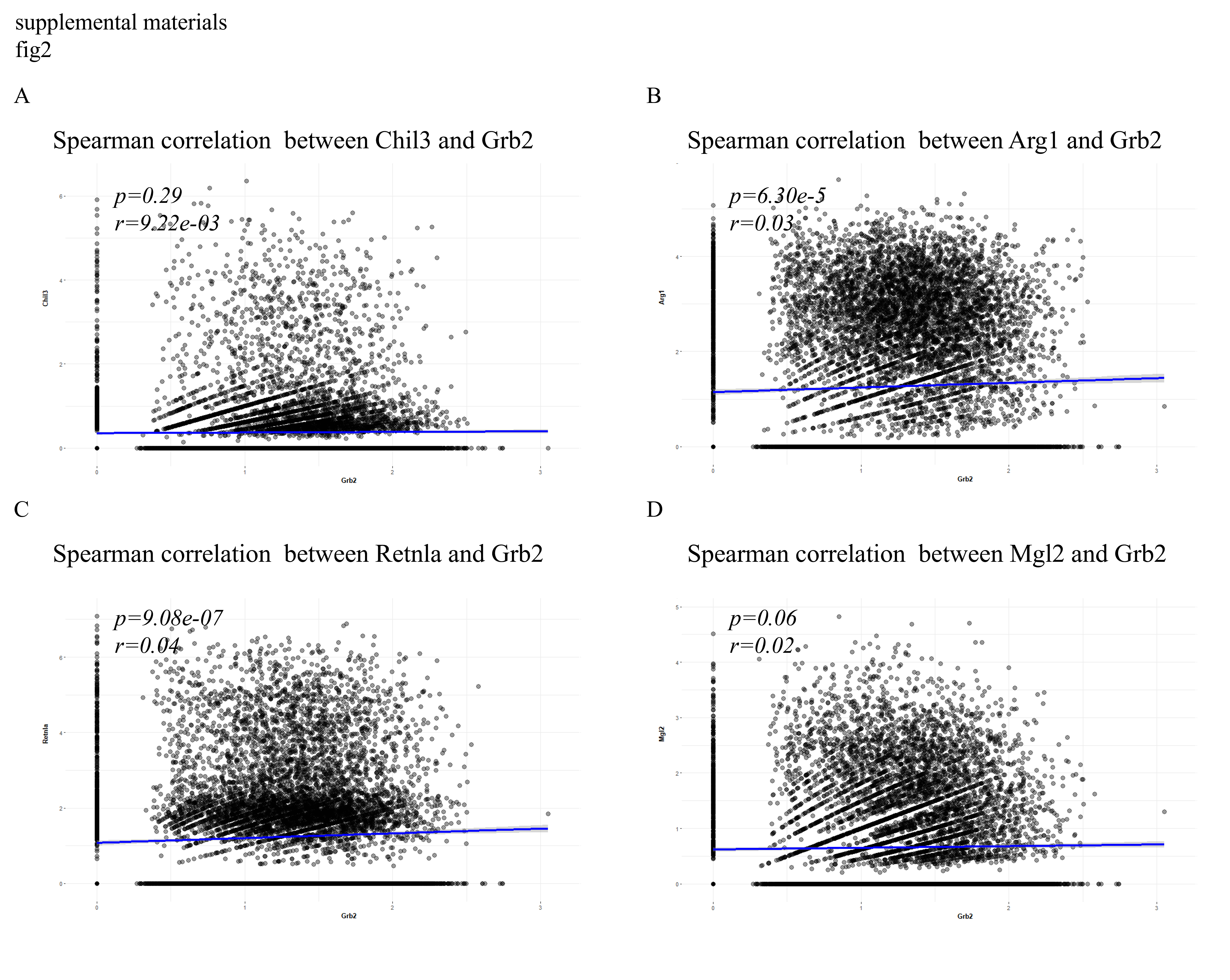

Supplement: Supplementary Figure 2 — Grb2 is not associated with M2 marker. (A) The Spearman correlation between Chil3 and Grb2. (B) The Spearman correlation between Arg1 and Grb2. (C) The Spearman correlation between Retnla and Grb2. (D) The Spearman correlation between Mgl2 and Grb2. [file Image2.tif]

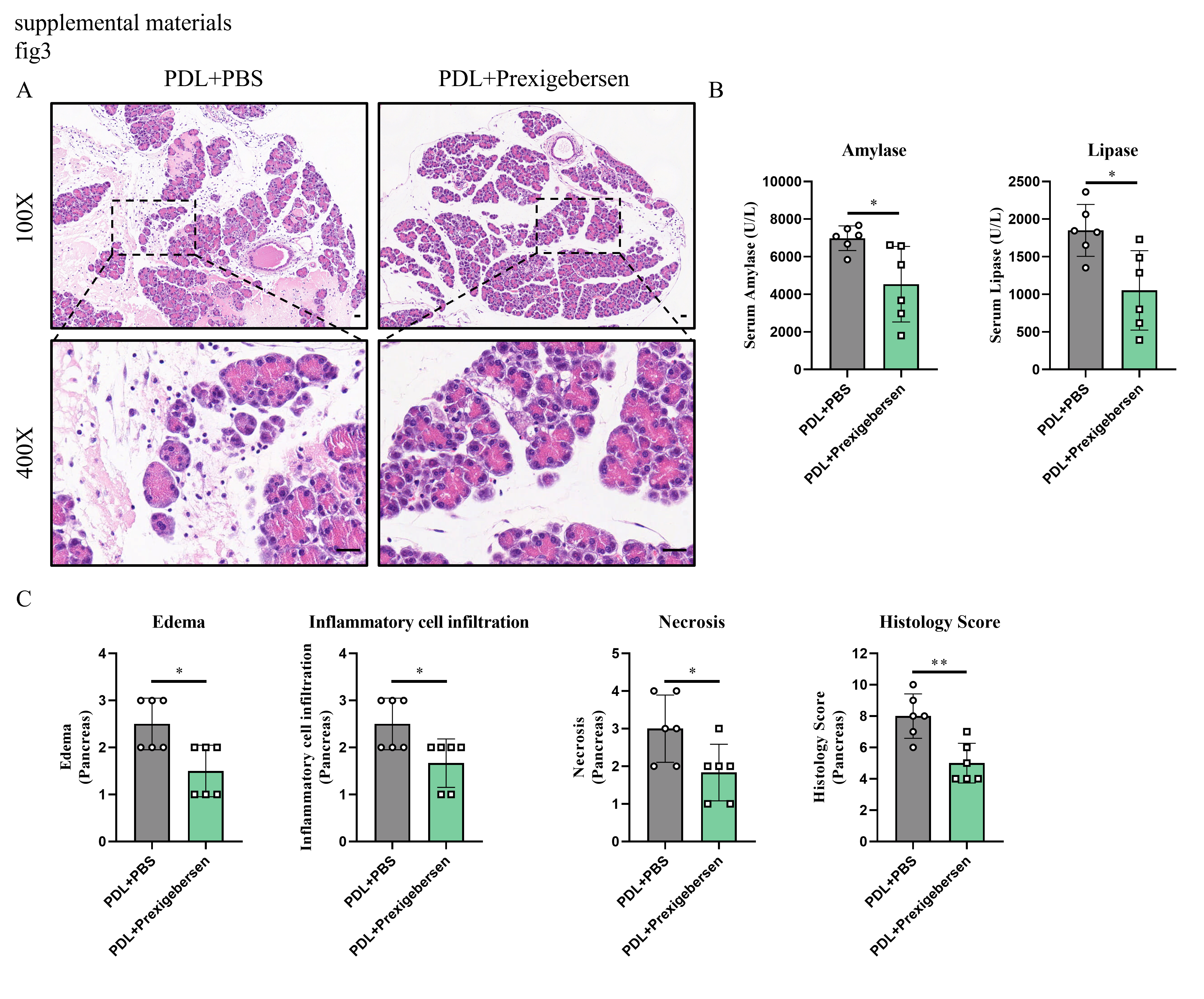

Supplement: Supplementary Figure 3 — Grb2 inhibitor alleviates PDL-induced AP. (A) Representative HE staining of pancreatic tissues in magnifications 100x and 400x. Scale Bar = 50μM. (B) Serum levels of amylase and lipase. (C) The pathological scores of pancreatic tissues. N=6 each group. N= 6 each group. *P < 0.05, **P < 0.01. [file Image3.tif]
